# Insight into the relationship between the cell culture model, cell trafficking and siRNA silencing efficiency

**DOI:** 10.1016/j.bbrc.2016.06.054

**Published:** 2016-08-19

**Authors:** Victoria Capel, Driton Vllasaliu, Peter Watts, Snow Stolnik

**Affiliations:** a Division of Drug Delivery and Tissue Engineering, School of Pharmacy, University of Nottingham, Nottingham, NG7 2RD, United Kingdom; b Archimedes Development Limited, Albert Einstein Centre, Nottingham Science and Technology Park, University Boulevard, Nottingham, NG7 2TN, United Kingdom

**Keywords:** Endocytosis pathways, Lung siRNA therapy, Pharmacological inhibitors, siRNA polyplexes, siRNA silencing

## Abstract

Despite research efforts, cell uptake processes determining siRNA silencing efficiency remain unclear. Here, we examine the relationship between *in vitro* cell culture models, cellular trafficking and siRNA silencing efficiency to provide a mechanistic insight on siRNA delivery system design. Model siRNA-polyplexes, based on chitosan as a ‘classical’ condensing agent, were applied to a panel of lung epithelial cell lines, H1299, A549 and Calu-3 and cell internalization levels, trafficking pathways and gene silencing assessed on exposure to pharmacological inhibitors. The data reveal striking differences in the internalization behaviour and gene silencing efficiency in the tested cell lines, despite their common lung epithelial origins. The model system’s silencing was lower where clathrin internalization pathway predominated in Calu-3, relative to silencing in H1299 cells where a non-clathrin internalization appears dominant. Increased silencing on endosomal disruption was apparent in Calu-3 cells, but absent when cellular internalization was *not* predominantly clathrin-mediated in A549 cells. This highlights that identifying cell trafficking pathways before incorporation of functional components to siRNA delivery systems (e.g. endosomolytic compounds) is crucial. The study hence stresses the importance of selection of appropriate cell culture model, relevant to *in vivo* target, to assess the gene silencing efficiency and decide which functionalities the ‘stratified siRNA silencing vector’ requires.

## Highlights

• Relationship between cell type, uptake path and silencing examined to inform siRNA vector design.
• Notable differences observed in cell uptake pathways and silencing despite cells’ common origin.
• Addition of endosomolytic functionality shows no effect when non-clathrin pathways dominate.
• Cell model important to assess silencing and decide which functionalities siRNA vector requires.

## 1 Introduction

Small interfering RNA (siRNA)-based therapeutics hold promise in the management of a wide variety of diseases, but their full potential rests on development of clinically suitable delivery carriers [1]. Understanding the cell entry processes of siRNA delivery systems is essential in enabling the prediction, design and optimization of transfection efficiency. The cell entry pathway of a delivery system is likely to affect intracellular processing and hence the silencing efficiency. Despite extensive research in developing siRNA delivery systems, understanding of the cell uptake mechanism of siRNA carriers is often inadequate, leading to an inability to predict or explain transfection efficiencies. This is particularly important as different materials have been proposed as siRNA-carrying systems, including polycations such as polyethylenimine [2], chitosan [3], dendrimers [4] and liposomes [5] and cell internalization and trafficking can be influenced by the nature of the carrier.

The present study uses an siRNA-complex based on a water soluble piperazine-modified chitosan [6] as a model system to investigate the relationship between gene silencing efficiency and cell internalization pathways in a panel of pulmonary epithelial cell lines, H1299, A549 and Calu-3. By employing well-established inhibitors of endocytosis processes in combination with cell imaging, we aimed to uncover differences in the internalization pathways and relate these to the systems’ silencing efficiency.

Pharmacological inhibitors were employed as a tool to decipher the mechanisms of siRNA-polyplex cell internalization under optimized working conditions. It is acknowledged that pharmacological inhibitors can exert multiple cellular effects, however, they are the most often used approach to study the mechanism of cellular trafficking [7], [11], [12], [13], [14], [15], [16] and the current understanding of nanoparticle trafficking is based on studies using pharmacological inhibitors. Recently, siRNA knock-down of endocytosis pathway-selective proteins has been advocated as an alternative [17], however this approach is still less adopted in the literature and has its own drawbacks [18]. As another alternative, we have recently applied a new microscopy approach to study co-localization with fluorescently labelled proteins of specific endocytosis pathways [19].

## 2 Materials and methods

Water-soluble piperazine chitosan derivatives were provided by Archimedes Pharma (UK). These were synthesised and characterized as described previously [6]. ‘DQ39’ derivative used in this study, N-[1-carboxymethyl-2-(1,4,4-trimethylpiperazi-1,4-dium)]chitosan dichloride, has an Mw of 24 kDa, Mn of 39.5 kDa, and degree of piperazine substitution (which renders the polymer soluble in water) of 39%.

GAPDH-specific siRNA was purchased from Dharmacon (CO, USA). Lipofectamine 2000 was purchased from Invitrogen (UK). All other chemicals, unless otherwise stated, were obtained from Sigma-Aldrich (UK). H1299, A549 and Calu-3 cell lines were purchased from the American Type Culture Collection (USA). H1299 cells were cultured in RPMI-1640 medium supplemented with 10% v/v foetal bovine serum (FBS), 1% v/v penicillin/streptomycin and 1% v/v l-glutamine. A549 and Calu-3 cells were cultured in Dulbecco’s Modified Eagle’s Medium (DMEM) and Eagle’s Minimal Essential Medium (EMEM) respectively, supplemented as above.

### 2.1 Formation of siRNA-polyplexes

Polyplexes were prepared in Tris-HCl buffer (10 mM, pH 7.4) by mixing (vortexing) equal volumes of DQ39 polymer and siRNA solutions to give a monomer:nucleotide ratio of 5:1 (unless otherwise stated, polyplexes were used at this ratio). After mixing, polyplexes were left at room temperature for 20 min before use.

### 2.2 In vitro silencing

Cells were seeded on 24-well plates (10^5^ cells/well) and cultured overnight to ∼70% confluency. siRNA-polyplexes (corresponding to 100 nM siRNA) were added to the cells in serum-free medium (HBSS:HEPES pH 7.4) and incubated for up to 4 h. Samples were then removed and replaced with fresh culture medium and cells cultured for 44 h. GAPDH activity was analysed using the KDalert GAPDH kit (Ambion, USA) following the supplier’s protocol, as a commonly used knock-down assay.

### 2.3 Cell uptake pathway analysis

Cells were seeded on 24-well plates (10^5^ cells/well) and cultured overnight to ∼70% confluency. To quantify siRNA internalization, siRNA-polyplexes containing Cy3-labelled siRNA (Termofisher Scientific, UK) (corresponding to 100 nM siRNA) were added to the cells in serum-free medium and incubated for 5, 15, 30, 60, 120, 180 or 240 min. To establish siRNA-polyplex internalization pathways, chemical inhibitors of endocytosis were employed (Supporting Information, Fig. S1). Cells were pre-incubated with inhibitors for 1 h at a concentration at which high viability was preserved in H1299 cells (Supporting Information, Fig. S2) followed by incubation with siRNA-polyplexes in serum-free medium containing the corresponding inhibitor. Cells were then washed with PBS and harvested by trypsin. Extracellular fluorescence of polyplexes associated with the cell surface (not internalized) was quenched with 0.04% v/v Trypan blue (in PBS) and cells were analysed immediately by flow cytometry using a Beckman Coulter Altra flow cytometer equipped with 488 nm and 556 nm lasers to obtain forward and side scatter and read Cy3, respectively. The emitted fluorescence light was collected using 585/23 nm band pass filter; 10,000 cells were analysed *per* sample. Data was analysed using Weasel Software Version 3.0.2 (The Walter and Eliza Hall Institute of Medical Research, Melbourne Australia).

Control experiments of clathrin and caveolae inhibition studies were conducted with known ligands for the clathrin and caveolae-mediated pathways (FITC-transferrin at 100 μg/ml and cholera toxin-B-subunit at 5 μg/ml, respectively) [7] (Supporting Information, Fig. S3).

### 2.4 Confocal microscopy

Cells were seeded in 24-well plates onto SecureSlip™ glass coverslips (Sigma-Aldrich, UK). Lysotracker™ Green DND-26 was applied to cells at 50 nM for 30 min. Cells were washed three times with PBS and fixed with 4% paraformaldehyde (PFA). Hoechst dye solution (100 μg/ml) was used for nuclei staining. Cell-containing coverslips were mounted (using DABCO mounting medium) onto glass slides for confocal imaging. Images were taken using a Leica TCS SP2 system mounted on a Leica DMIRE2 inverted microscope.

### 2.5 Statistical analysis

Statistical comparisons for more than two data groups employed one way analysis of variance (ANOVA) followed by Bonferroni post-hoc test, while comparisons of two data groups were performed using Student’s *t*-test. Values of *p* < 0.05 were considered statistically significant.

## 3 Results

**Fig. 1:**
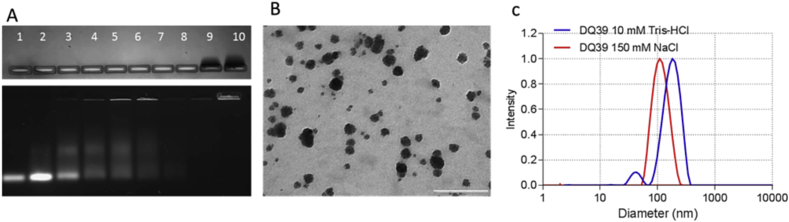
Physicochemical properties of model siRNA-polyplexes (DQ39-siRNA). A) Agarose gel electrophoresis. Lane 1: siRNA control; lanes 2–9: siRNA-polyplexes at monomer:nucleotide ratios of 0.25, 0.5, 0.75, 1.0, 1.25, 1.5, 2.0 and 5.0:1; lane 10: polymer control. B) Transmission electron microscopy; scale 1000 nm. C) Hydrodynamic diameter distribution, measured by dynamic light scattering of siRNA-polyplexes in low or physiological ionic strength buffers (pH 7.4), Tris-HCl 10 mM or NaCl 150 mM.

Fig. 1 summarizes the physicochemical properties of model siRNA-polyplexes, prepared at 5:1 polymer monomer:siRNA nucleotide ratio to ensure a system with good colloidal stability in a physiological ionic strength buffer (Fig. 1C) and with minimal presence of free condensing polymer (Fig. 1A, upper panel). Gel electrophoresis indicates that siRNA complexation occurs from low monomer:nucleotide ratios, observed by the fading of free siRNA bands from a 0.25:1 ratio onwards and the absence of free siRNA at 5:1 ratio (lower panel, Fig. 1A). Polyplexes possess an average diameter of ∼150 nm and surface charge of +30 mV, which are typical features of siRNA delivery systems [8], [9], [10].

### 3.1 Gene silencing and cellular uptake profiles

**Fig. 2:**
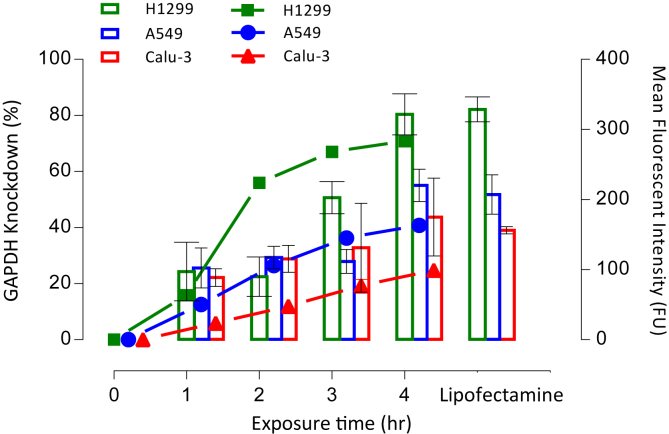
siRNA polyplex internalization (line) and GAPDH silencing (bars) with time in a panel of lung epithelial cell lines. Polyplexes were applied in serum-free HBSS:HEPES medium. Cell internalization was assessed by flow cytometry of Cy3-siRNA-polyplexes; minimum 10,000 cells were analysed per sample. GAPDH activity measurements were conducted in cells incubated in growth medium for 44 h following complex addition and removal. Statistical comparison for uptake: A549 *vs* Calu-3: p < 0.0001 at all time points; H1299 *vs* A549: p < 0.05 at 1 h and p < 0.0001 at all other time points. Statistical comparison for knockdown: A549 *vs* Calu-3: non-significant for 1–3 h time points and p < 0.05 at 4 h. H1299 *vs* A549: non-significant for 1 and 2 h time points and p < 0.0001 for 3 and 4 h.

Gene silencing efficiencies of model siRNA-polyplexes were evaluated and compared to their cell internalization level at corresponding time points (Fig. 2). There is a clear time effect in all cell lines; longer exposure to siRNA-polyplexes resulted in an increased cell internalization and silencing. There are no dramatic differences in the *silencing* between tested cell lines at one and two hours exposure, whilst the values for polyplex *internalization* for H1299 are significantly higher compared to A549 and Calu-3 cells at the two-hour time point, illustrating differences in polyplex uptake between cells types. Silencing effects and internalization levels at four hours exposure show significant differences between the cells, with 75% knockdown for H1299 cells, 55% for A549 and 43% for Calu-3. Significant cell type effect on the silencing levels is also seen for Lipofectamine, with similar overall silencing to the model chitosan system.

**Fig. 3:**
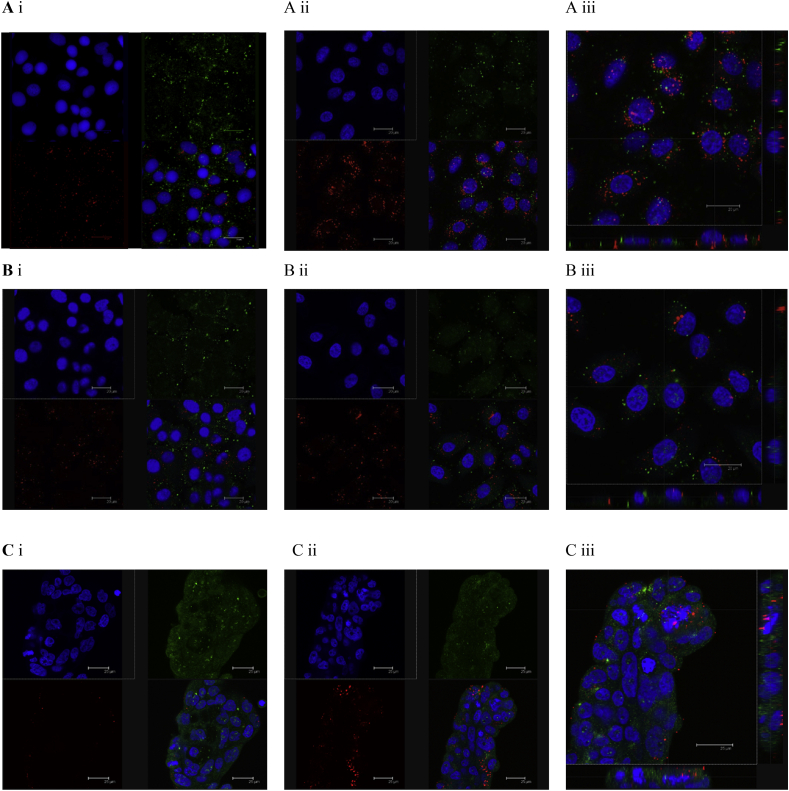
Confocal microscopy images of siRNA-polyplex internalization in A) H1299, B) A549 and C) Calu-3 cells. Cy3-labelled siRNA (red) complexes with DQ39 at 5:1 monomer:nucleotide ratio were incubated with cells for i) 1 h or ii) 4 h iii) z-stack of siRNA-polyplexes internalization at 4 h. Nuclei appear in blue, lysosomal compartments stained with LysoTracker Green (green). Scale bar: 20 μm (A and B) and 25 μm (C). (For interpretation of the references to colour in this figure legend, the reader is referred to the web version of this article.)

In addition to flow cytometry, confocal microscopy was also employed with a lysosomal marker to assess polyplex cell uptake following 1 and 4 h exposure. Micrographs in Fig. 3A–C suggest that in H1299 cells the level of polyplex-associated fluorescence appears higher relative to A549 and Calu-3 cells, in line with measured cell internalization in Fig. 2. Polyplex florescence appears dispersed intracellularly, within vesicular compartments. Fig. 3A indicates a high level of polyplex-associated florescence (red puncta), whereby the spatial arrangement is different to the lysosomal marker (green). This suggests that polyplexes are predominantly distributed in the cytosol and not associated with the lysosomes. In A549 cells, polyplex fluorescence (Fig. 3B) is lower relative to H1229 cells, which corroborates with uptake study data in Fig. 2. The spatial arrangement of polyplex and lysosome-associated fluorescence again indicates that polyplexes do not co-locate with the lysosomes. With Calu-3 cells (Fig. 3 Ci-iii), growth on glass substrate as ‘cell islands’ (despite sub-confluence) makes the interpretation of confocal microscopy data difficult.

### 3.2 Cell internalization pathways

**Fig. 4:**
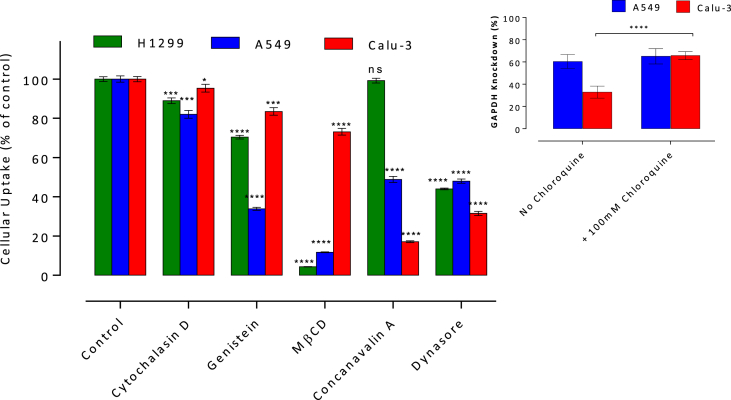
Cell internalization of siRNA-polyplexes in the presence of pharmacological endocytosis inhibitors. Inhibitors were applied to the cells for 1 h before polyplex addition (corresponding to 100 nM siRNA). Cells were treated with polyplexes for 2 h in serum-free medium containing concanavalin-A 100 μg/ml, dynasore 100 μM, genistein 100 μM, MβCD 4 mM, cytochalasin D 2 μg/ml. Comparisons relative to control, untreated cells. Inset: GAPDH knockdown with siRNA-polyplexes in A549 and Calu-3 cells in the presence of chloroquine. Polyplexes were incubated with the cells in serum-free medium or medium containing chloroquine for 4 h, with further 44 h incubation in medium before analysis. Data shown as the mean ± SD (n = 3 inhibitor study and n = 6 chloroquine study). ‘ns’ not significant; * p < 0.05; *** p < 0.001,**** p < 0.0001.

Fig. 4 summarizes the effects of endocytosis inhibitors on cell internalization of siRNA-polyplexes and reveals clear cell type-dependent differences. In H1299 cells, treatments with caveolae pathway inhibitors, genistein or MβCD, resulted in a statistically significant reduction of siRNA-complex uptake, relative to the untreated control, whilst concavalin-A (clathrin pathway inhibitor) did not exert a statistically significant effect. Interestingly, genistein and MβCD effects are notably different. Genistein blocks caveolae-dependent endocytosis by acting as an inhibitor of tyrosine kinase, whilst MβCD is a sterol-binding compound that sequesters cholesterol from the cell membrane, affecting lipid rafts and caveolae-dependent pathway (Supporting Information, S1), hence affecting the caveolar pathway by different mechanisms. The reasons for genistein-MβCD apparent discrepancy are not clear presently. We, however, noticed a relatively moderate effect of genistein on H1299 internalization of the caveolae pathway specific ligand, cholera toxin B, (Supporting Information, Fig. S3B).

In A549 cells, both clathrin and caveolae endocytosis inhibitors display significant effects: concavalin-A reduces cell internalization to approximately 50% of the untreated control, with caveolae-dependent pathway inhibition by genistein and exposure to MβCD reducing internalization to approximately 35% and 10%, respectively. Calu-3 cells exhibited a marked reduction in polyplex uptake (approximately 20% of untreated control) on inhibition of the clathrin pathway (concavalin-A treatment), whilst genistein or MβCD inhibition of caveolae-dependent pathway suppressed the uptake to a significantly lesser extent (to around 80% and 70% of the untreated control, respectively). Polyplex cell uptake in the presence of dynasore, an inhibitor of both clathrin and caveolae-mediated endocytosis, is reduced to 40–50% of untreated control in all cell lines, while cytochalasin D, an inhibitor of macropinocytosis, displays a small, although statistically significant effect.

Next, we investigated whether the relatively low levels of silencing in A549 and Calu-3 cells, are a consequence of the nature of endocytosis that predominately involves the clathrin route. To this end, gene silencing was conducted in the presence of the endosomolytic agent, chloroquine (Fig. 4, inset). In Calu-3 cells chloroquine treatment significantly increased the silencing efficiency, from 33% to 66% of the control, whereas no significant influence was observed in A549 cells. Data thus indirectly point to a principally clathrin-mediated internalization of siRNA polyplexes by Calu-3 cells, whereby the silencing efficiency is enhanced by the endosomolytic action of chloroquine.

## 4 Discussion

Effective siRNA delivery into target cells is the main barrier to translation of siRNA therapeutics into the clinic. Cell trafficking pathways of siRNA are still not clearly understood to inform the formulation development [15], [20]. In this study, we investigated the mechanisms by which siRNA-polyplexes enter different types of lung-derived epithelial cells, as means of probing the potential relationship between cell entry pathways and silencing efficiency.

Of the selected lung epithelial cell lines, H1299 cultures are often used to report siRNA silencing for lung application systems (considered ‘suitable transfection hosts’) [8], [21], [22]. Calu-3 and A549 cell lines are significantly less utilised in these studies, despite appreciation that they offer a more representative model of the airway epithelium [23], [24], [25]. The lung is a prominent target for local, non-invasive siRNA delivery for lung cancers and other diseases such as chronic obstructive pulmonary disease, asthma and respiratory syncytial virus [26], [27], [28], highlighting the relevance of this study.

Our data demonstrate a cell type-dependent level of siRNA-polyplex internalization, trafficking and silencing efficiency in different cells. The literature highlights a complex relationship between siRNA polyplex cell uptake and silencing efficiency [20] and the data in Fig. 2 confirms this complexity.

Contribution of both clathrin and caveolin-dependent pathways in the internalization of siRNA-polyplexes is apparent in the A549 cell line, in line with our previous study for DNA-polyplexes, which also showed involvement of microtubules and actin filaments (confirming vesicular intracellular transport) [29]. The data rules out a prominent role of dynamin-independent endocytosis (macropinocytosis) in all tested epithelial cell types, as judged from the small reduction in polyplex cell uptake following treatment with cytochalasin D (∼10%) and substantial (∼50%) reduction with dynasore, an inhibitor that plays an essential role in the ‘release’ of the formed caveolin- and clathrin-coated vesicles [30]. This is in disagreement with a recent study in HeLa cells whereby down-regulation of macropinocytosis components (Cdc42 and Rac1) decreased the internalization of siRNA-loaded cationic lipid nanoparticles by ∼80%, whereas inhibition of clathrin and caveolae-dependent pathways had little impact on cell entry [15]. However, another recent study utilizing HeLa cells reported a significant reduction in cell uptake of DNA-loaded lipid nanoparticles following inhibition of clathrin-mediated and clathrin-independent endocytosis, suggesting the involvement of vesicle-mediated internalization [31]. In the DNA-delivery field, a study comparing the transfection efficiency of chitosan-alginate polyplexes in diverse cell types reported that the uptake route is dependent on the cell line and has significant consequences on gene transfection efficiencies. Cell uptake by the clathrin pathway was suggested to be responsible for successful gene expression in 293T and Cos7 cells (due to the ‘proton sponge’ endosomal escape), whilst caveolin-mediated endocytosis in CHO cells resulted in vesicle-entrapped polyplexes that become transfection-incompetent [32].

In Calu-3 cells, clathrin pathway inhibition by concavalin-A resulted in notable reduction of polyplex uptake (∼80%), while a much lower effect was apparent on inhibition of caveolae-dependent pathway (∼20% and ∼30% reduction for genistein and MβCD, respectively) – markedly lower compared to A549 and H1299 cells, therefore pointing to a considerable involvement of non-clathrin/caveolin internalization. Lysosomal disruption in Calu-3 cells enhanced gene silencing, an observation not apparent in A549 cells. The data therefore suggests that piperazine-substituted chitosans follow a predominantly clathrin-mediated entry route in Calu-3 cells and may not demonstrate an efficient endosomal escape. Our findings also imply that the incorporation of an endosomolytic component into the siRNA delivery system where the dominant cellular internalization pathway involves clathrin may be beneficial to augment the silencing efficiency, but such functionality could be purposeless in non-clathrin cell internalization pathways.

This study shows that model polyplexes exhibited the highest silencing effect in H1299 cells, whereby a predominantly caveolin-mediated internalization was apparent. Interestingly, in control experiments we noticed that transferrin internalization (clathrin pathway marker) by H1299 cells was relatively low, in both extent and rate (Supporting Information, Fig. S3). H1299 cells are reported to have a homozygous partial deletion of the TP53 gene and do not express the tumour suppressor p53 protein [33]. The most commonly used cell lines in siRNA transfection studies, such as H1299, HeLa and CHO cells, all considered as ‘suitable transfection hosts’, have either low or non-functioning p53 expression [34], [35], whereas A549 and Calu-3 cell lines, as more representative culture models of the ‘normal’ lung, both express wild type p53 [36], [37]. There is a suggestion that the p53 protein is involved in the regulation of clathrin endocytosis; p53 knockdown delays the uptake of epidermal growth factor receptor (EGFR) – one of the most characterized receptors to study the mechanisms of clathrin-mediated endocytosis [38]. Further studies are required to confirm the p53-clathrin connection, however if cell lines with low or non-functioning p53 expression indeed lack effective clathrin-mediated endocytosis, with ‘alternative’ non-clathrin uptake pathways assuming a predominant function, this would be an important parameter to understand in relation to the design of siRNA delivery systems. Furthermore, establishing its relevance to the *in vivo* situation and lung cancer models [39] would be advantageous for *in vitro – in vivo* data translation.

Overall, our study highlights the importance of cell model selection, even in closely related cell types, on understanding of the silencing efficacy of siRNA delivery systems. Furthermore, it emphasises that knowledge on cell trafficking processes is essential in deciding the design parameters of siRNA delivery systems.
