# Supplementary material for: Insight into the relationship between the cell culture model, cell trafficking and siRNA silencing efficiency
Source: Biochem Biophys Res Commun. 2016 Aug 19;477(2):260–5. doi: 10.1016/j.bbrc.2016.06.054 (PMC4948577; doi:10.1016/j.bbrc.2016.06.054)
Supplement: Supplementary file 1 [file mmc1.doc]

**Supplementary Information**

Insight into the relationship between the cell culture model, cell trafficking and siRNA silencing efficiency

by Victoria Capel, Driton Vllasaliu, Peter Watts and Snow Stolnik


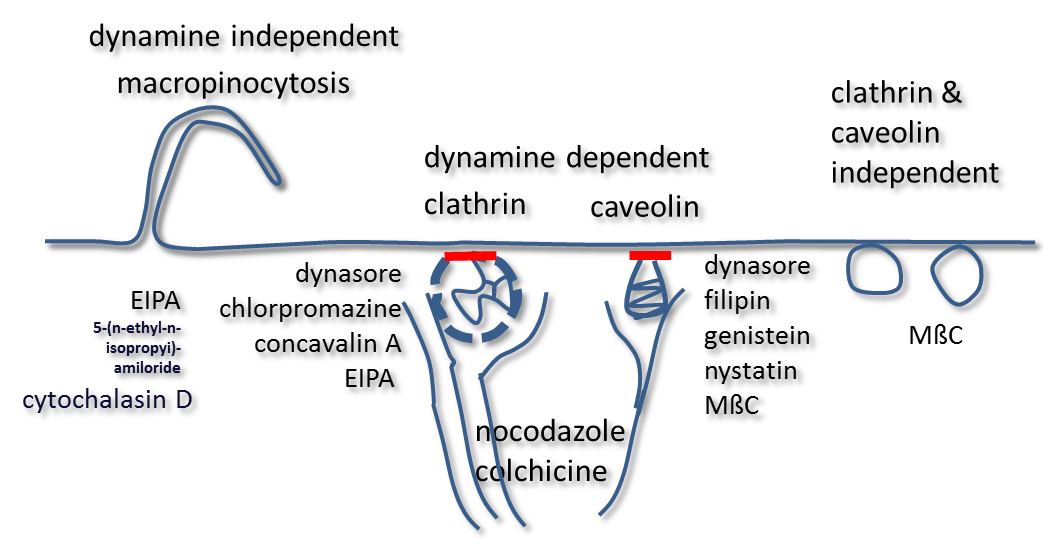


| **Inhibitor** | **Pathway** | **Mechanism** | **Ref** |
| --- | --- | --- | --- |
| Dynasore | Clathrin and caveolae endocytosis | Inhibitor of dynamin, prevents scission of endocytic vesicles | [1] |
| Chlorpromazine | Clathrin endocytosis | Causes translocation of clathrin from membrane to intracellular vesicles | [2] |
| Concanavalin A | Clathrin endocytosis | Prevents formation of clathrin coated pits | [3] |
| Nystatin | Caveolae endocytosis | Sequesters membrane cholesterol | [4] |
| Genestein | Caveolae endocytosis | Tyrosine kinase inhibitor | [5] |
| Filipin | Caveolae endocytosis | Binds to membrane cholesterol | [6] |
| Methyl-β-Cyclodextrin | Lipid raft endocytosis / Caveolae / Macropinocytosis | Depletes membrane cholesterol | [7] |
| Colchicine | Microtubules | Microtubule depolymerisation | [8] |
| Nocodazole | Microtubules | Microtubule depolymerisation | [9] |
| Cytochalasin D | Macropinocytosis | Actin depolymerisation | [10] |
| Chloroquine | Disruption of endosomes/lysosomes | Prevents acidification of endosomes | [11] |

**Figure S1. Chemical inhibitors of endocytosis and their mechanism of action (with illustration).**

**Figure S2. Cell toxicity of endocytosis inhibitors.** H1299 cells were incubated with inhibitors for 3 hours. Metabolic activity was measured using an MTS assay and compared to controls (cells in medium taken as 100% viability and in Triton X as 0 % viability. Data is shown as the mean +/- SD (n=8).

**A)**

**
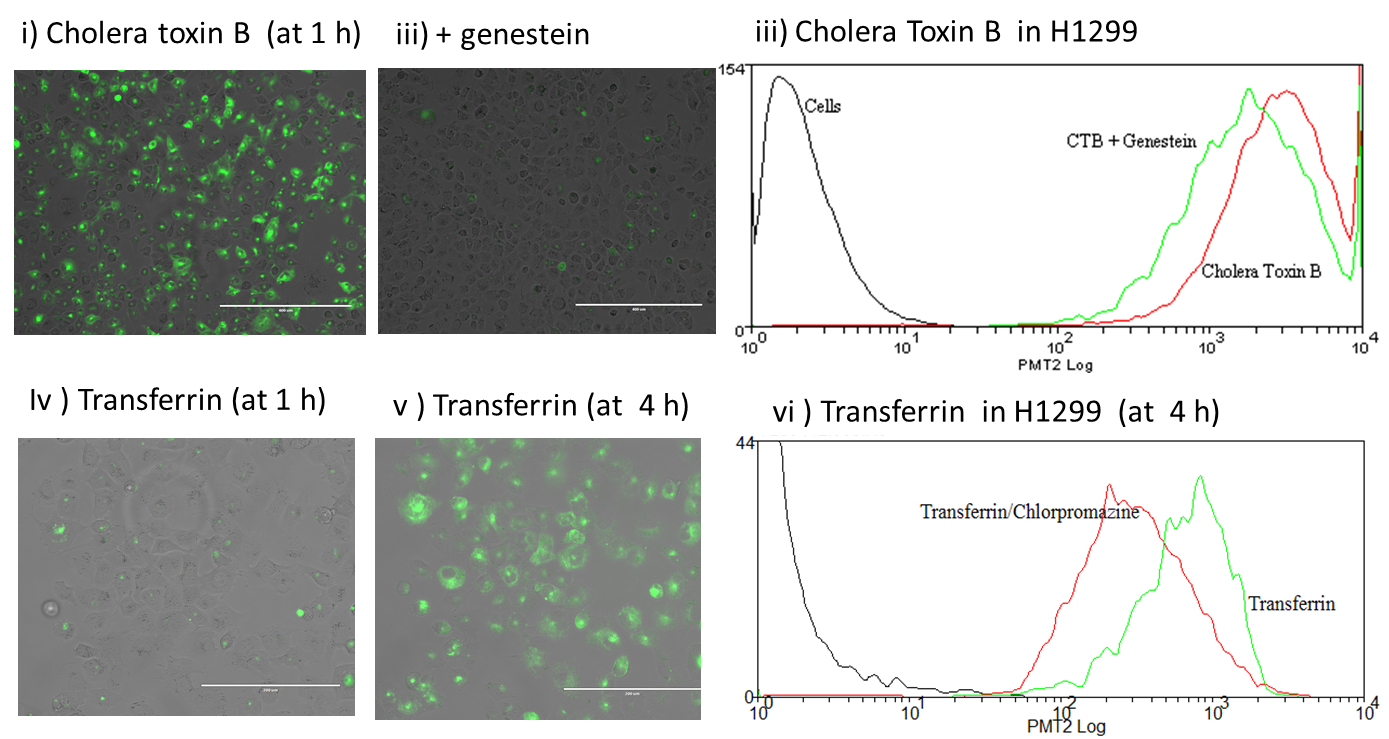
**

**B)**

**i ii**

**Figure S3. Internalization of pathway-specific ligands in H1299 cells and effect of endocytosis inhibitors. A**) Fluorescence microscopy images for internalization of FITC labelled (green) Cholera Toxin B (1 µg/ml) at 1 hour post application in i) absence or ii) presence of genistein (100 µM) inhibitor. iii) Flow cytometry analysis at 1 hour in absence and presence of genistein inhibitor. Fluorescent microscopy images of FITC labelled (green) transferrin internalization by H1299 cells at iv) 1 hour of exposure and v) 4 hours of exposure. Concentration of transferrin applied 50 µg/ml. vi) Flow cytometry analysis at 4 hours in absence and presence of chlorpromazine inhibitor. Scale bar 200 µm. **B**) uptake of FITC-transferrin in the presence of concanavalin A (100 µg/ml) (i) and uptake of FITC-cholera toxin B in the presence of genestein (100 µM) (ii). Data is shown as the mean +/- SD, *** p<0.001, **** p<0.0001.

**References**

[1] T. Kirchhausen, E. Macia, H.E. Pelish, Use of dynasore, the small molecule inhibitor of dynamin, in the regulation of endocytosis., Methods Enzymol. 438 (2008) 77–93.

[2] Z.M. Qian, H. Li, H. Sun, K. Ho, Targeted drug delivery via the transferrin receptor-mediated endocytosis pathway. Pharmacol. Rev. 54 (2002) 561–87.

[3] H. Tang, Inhibition of AT1 Receptor Internalization by Concanavalin A Blocks Angiotensin II-induced ERK Activation in Vascular Smooth Muscle Cells. INVOLVEMENT OF EPIDERMAL GROWTH FACTOR RECEPTOR PROTEOLYSIS BUT NOT AT1 RECEPTOR INTERNALIZATION, J. Biol. Chem. 275 (2000) 13420–13426.

[4] Y. Chen, S. Wang, X. Lu, H. Zhang, Y. Fu, Y. Luo, Cholesterol sequestration by nystatin enhances the uptake and activity of endostatin in endothelium via regulating distinct endocytic pathways., Blood. 117 (2011) 6392–403.

[5] T. Akiyama, J. Ishida, S. Nakagawa, H. Ogawara, S. Watanabe, N. Itoh, et al., Genistein, a specific inhibitor of tyrosine-specific protein kinases., J. Biol. Chem. 262 (1987) 5592–5.

[6] J.E. Schnitzer, P. Oh, E. Pinney, J. Allard, Filipin-sensitive caveolae-mediated transport in endothelium: reduced transcytosis, scavenger endocytosis, and capillary permeability of select macromolecules., J. Cell Biol. 127 (1994) 1217–32.

[7] A. Christian, M. Haynes, M. Phillips, G. Rothblat, Use of cyclodextrins for manipulating cellular cholesterol content, J. Lipid Res. 38 (1997) 2264–2272.

[8] D.A. Skoufias, L. Wilson, Mechanism of inhibition of microtubule polymerization by colchicine: inhibitory potencies of unliganded colchicine and tubulin-colchicine complexes., Biochemistry. 31 (1992) 738–46.

[9] J.S. Goltz, A.W. Wolkoff, P.M. Novikoff, R.J. Stockert, P. Satir, A role for microtubules in sorting endocytic vesicles in rat hepatocytes., Proc. Natl. Acad. Sci. U. S. A. 89 (1992) 7026–30.

[10] T. Wakatsuki, B. Schwab, N.C. Thompson, E.L. Elson, Effects of cytochalasin D and latrunculin B on mechanical properties of cells., J. Cell Sci. 114 (2001) 1025–36.

[11] R. Wattiaux, N. Laurent, S. Wattiaux-De Coninck, M. Jadot, Endosomes, lysosomes: their implication in gene transfer, Adv. Drug Deliv. Rev. 41 (2000) 201–208.
